# Exogenous rhTRX reduces lipid accumulation under LPS-induced inflammation

**DOI:** 10.1038/emm.2013.136

**Published:** 2014-01-10

**Authors:** Gi-Yeon Han, Eun-Kyung Lee, Hey-won Park, Hyun-Jung Kim, Chan-Wha Kim

**Affiliations:** 1 College of Life Sciences and Biotechnology, Korea University, Seoul, Korea

**Keywords:** inflammation, lipid accumulation, rhTRX, skin cell proteomics, TIP47

## Abstract

Redox-regulating molecule, recombinant human thioredoxin (rhTRX) which shows anti-inflammatory, and anti-oxidative effects against lipopolysaccharide (LPS)-stimulated inflammation and regulate protein expression levels. LPS-induced reactive oxygen intermediates (ROI) and NO production were inhibited by exogenous rhTRX. We identified up/downregulated intracellular proteins under the LPS-treated condition in exogenous rhTRX-treated A375 cells compared with non-LPS-treated cells via 2-DE proteomic analysis. Also, we quantitatively measured cytokines of *in vivo* mouse inflammation models using cytometry bead array. Exogenous rhTRX inhibited LPS-stimulated production of ROI and NO levels. TIP47 and ATP synthase may influence the inflammation-related lipid accumulation by affecting lipid metabolism. The modulation of skin redox environments during inflammation is most likely to prevent alterations in lipid metabolism through upregulation of TIP47 and ATP synthase and downregulation of inflammatory cytokines. Our results demonstrate that exogenous rhTRX has anti-inflammatory properties and intracellular regulatory activity *in vivo* and *in vitro*. Monitoring of LPS-stimulated pro-inflammatory conditions treated with rhTRX in A375 cells could be useful for diagnosis and follow-up of inflammation reduction related with candidate proteins. These results have a therapeutic role in skin inflammation therapy.

## Introduction

Skin, the largest organ of the human body, is the most important interface between the environment and the body. It is constantly exposed to chemical and physical environmental pollutants or their metabolic toxicants, which are associated with a wide range of inflammatory skin diseases. Inflammatory skin diseases, such as dermatitis, systemic inflammatory response syndrome (SIRS) and sepsis, are known to affect the whole body, which results in systemic inflammation, organ dysfunction and organ failure.^1^ Recent studies have reported that oxidative stress has a critical role in the upregulation of local inflammatory mediators.^2^ Moreover, severe depletion of antioxidants in the skin caused by prolonged exposure to reactive oxygen species (ROS) results in insufficient protection, triggering cellular damage.^3, 4, 5, 6^ Thus, reactive oxygen intermediates (ROI) have emerged as promising targets for anti-inflammatory drug discovery. Topical application or oral administration of antioxidants has been suggested as an effective preventive therapy for inflammatory skin diseases.

Thioredoxin (TRX) is a multifunctional thiol molecule with antioxidant, anti-inflammatory and antiapoptotic properties.^7, 8^ It functions as a general oxidoreductase with a conserved CXXC active site that forms a disulfide in the oxidized form and a dithiol in the reduced form.^9, 10, 11, 12^ These two cysteines are the key for the ability of TRX to facilitate the reduction of other proteins and maintain cellular redox homeostasis.^13, 14, 15, 16, 17, 18^ TRX levels have been shown to be related with organism lifespan and age-associated tissue deterioration.^18^ Recent studies have reported that TRX has protective effects against various inflammatory diseases.^2^ In addition, TRX expression is enhanced under various inflammatory conditions.^8, 19^ TRX treatment decreases not only oxidative stress but also the inflammatory mediator NO.^20^ Accumulated evidence has also suggested that the administration of recombinant redox-regulating molecule recombinant human thioredoxin (rhTRX) induces increased tolerance against oxidative stress and inflammation.^21, 22^ However, despite recent progress in TRX research in terms of inflammation, the protective effects and action mechanisms against skin inflammation have not yet been entirely elucidated. Therefore, this study was conducted to evaluate the protective effects of exogenous rhTRX on LPS-stimulated skin cells. To determine this, we used proteomic techniques in the belief that large-scale analyses via proteomic approaches might help to systematically understand the action mechanisms of rhTRX on inflammatory skin diseases. In addition, as a skin inflammation model, the human melanocyte cell line, A375 melanoma, as well as C57BL/6 mice, was used. As only a few studies have examined this phenomenon, the results of this study would also help broaden our understanding of inflammatory damage in melanocytes. Here we report that exogenous rhTRX regulates inflammatory skin conditions, especially through its ability to modulate lipid metabolism by prompting the expression of related proteins TIP47 and ATP synthase and by decreasing TNF-α, MCP-1 and IL-6. Elucidation of the mechanism underlying the anti-inflammatory function of exogenous rhTRX might contribute to the discovery of new therapeutic targets for the treatment of skin inflammation-related diseases.

## Materials and methods

### Purification of rhTRX

rhTRX was purified from *Escherichia coli* BL21 (DE3) pLysS transformed with pET28a-6His-rhTRX. The expression vector encoded the full-length rhTRX protein, which is nearly identical to the human TRX-1, fused to a polyhistidine tag at its NH_2_ terminus. Protein expression and purification were conducted under native conditions using Ni-NTA resin (Qiagen, Valencia, CA, USA) following the manufacturer's recommendations. Residual RNA and DNA were removed from the column by incubating the resin with a buffer containing 50 mM of NaH_2_PO_4_, 300 mM of NaCl, 10 mM of imidazole, 10 mM of β-mercaptoethanol, pH 8.0, DNase (Promega, Madison, WI, USA; 1.5 μg ml^−1^) and RNase A (15 μg ml^−1^) for 2 h at 25 °C with continuous shaking. rhTRX was then eluted with a buffer containing 50 mM of NaH_2_PO_4_, 300 mM of NaCl, 50 mM of imidazole and 10 mM of β-mercaptoethanol, pH 8.0. Residual bacterial LPS was extracted using Triton X-114. Briefly, 1/20 (v/v) Triton X-114 was added to the solution containing the recombinant protein. The mixture was incubated for 1 h at 4 °C with constant rotation for 20 min at 37 °C. The sample was then centrifuged at 4500 *g* for 15 min. The supernatant was collected and extensively dialyzed against PBS. Residual bacterial LPS was measured with the E-toxate reagent (Sigma, St Louis, MO, USA) according to the manufacturer's instructions.^23^

### Cell culture and reagents

The human melanoma cell line A375 melanoma was purchased from the American Type Culture Collection (Rockville, MA, USA, CRL-1619). A375 cells were cultivated at 37 °C in a humidified incubator supplied with 5% CO_2_. The medium consisted of DMEM supplemented with 10% of fetal bovine serum, penicillin (100 U ml^−1^) and streptomycin (100 μg ml^−1^). When the A375 cells were 80% confluent, they were treated with bacterial LPS (10 μg ml^−1^). Medium and other cell culture reagents were obtained from Gibco-BRL (Grand Island, NE, USA). Precast IPG strips and other reagents used in 2-DE experiments were from Amersham Biosciences (Uppsala, Sweden). Antibodies were from Santa Cruz Biotechnology (Santa Cruz, CA, USA).

### Detection of ROIs and NO

Intracellular ROI production was measured by following the method described by Hyun *et al.*^24^ Briefly, A375 melanoma cells (1 × 10^4^ cells/well) were placed in a 96-well plate and activated with 10 μg ml^−1^ of bacterial LPS (*E. coli* serotype 0111:B4; Sigma). After 4 h, the cells were treated with exogenous rhTRX and then incubated with 50 μM of 2′, 7′-dichlorodihydrofluorescein diacetate (DCFH-DA; Sigma). ROI generation was analyzed using a Multilabel Plate Reader (Perkin Elmer, MA, USA) with excitation at 485 nm and emission at 530 nm.

Nitrite formation was determined using the Griess assay according to the manufacturer's instructions (Promega, Heidelberg, Germany). A375 melanoma cells were seeded at a density of 1 × 10^5^ cells per well in 96-well plates. After incubation for 12 h, cells were incubated with 10 μg ml^−1^ of LPS for 4 h. Thereafter, medium was changed and the cells were further incubated for 4 h with or without the addition of 50 μg ml^−1^ of rhTRX. Nitrite concentrations in the supernatant of A375 melanoma cells were calculated in comparison with standard concentrations of NaNO_2_ dissolved in culture medium or PBS. Absorbance was read at 540 nm, and nitrite concentrations were calculated.

### 2-DE and image analysis

2-DE samples were prepared according to the previously described methods.^25, 26, 27, 28, 29^ After delipidation and desalting, the protein concentration of the samples was measured via a modified Bradford method using BSA as a standard.^30^ Immobilized DryStrips (24 cm, pH 3-10) utilized for isoelectric focusing (IEF) were rehydrated with 40 μg of protein in 450 μl of solubilization solution containing 8 M of urea, 2% of CHAPS, 1% of immobilized pH gradient (IPG) buffer (pH 3-10), 13 mM of dithiothreitol (DTT) and a trace of bromophenol blue for 5 h without current and for another 7 h at 80 V. IEF was conducted using the IPGphor IEF system (GE Healthcare, Uppsala, Sweden) for 120 000 Vhr. The second dimension was run on 12.5% of SDS-PAGE with an Ettan DALT II system (GE Healthcare). Proteins were visualized *via* silver staining. All experiments were conducted in triplicate. Computer analyses of the 2-DE images were conducted using an ImageMaster 2D Elite Software (GE Healthcare). The expression levels of the spots were determined in accordance with the relative spot volume of each protein, as compared with the normalized volumes of proteins.^31^

### Protein identification by ESI Q-TOF MS/MS

Excised gel spots were destained using 100 μl of destaining solution (1:1=30 mM potassium ferricyanide: 100 mM sodium thiosulfate, v/v) for 5 min with agitation. After removal of the solution, the gel spots were incubated for 20 min with 200 mM of ammonium bicarbonate. The gel pieces were then dehydrated with 100 μl of acetonitrile and dried in a vacuum centrifuge. The dried gel pieces were rehydrated with 20 μl of 50 mM ammonium bicarbonate containing 0.2 μg of modified trypsin (Promega Corp., WI, USA) on ice for 45 min. After the removal of the solution, 30 μl of 50 mM ammonium bicarbonate was added. The digestion was performed overnight at 37 °C. The peptide solution was desalted using a ZipTip_C18_ nano column (Millipore Corp., Bedford, MA, USA). Thirty microliters of the peptide mixture from the digestion supernatant was diluted in 30 μl of 5% formic acid, loaded onto the column and then washed with 30 μl of 5% formic acid. For MS/MS analysis, the peptides were eluted with 1.5 μl of 50% methanol, 49% H_2_O and 1% formic acid directly into a precoated borosilicate nanoelectrospray needle (Micromass, Manchester, UK).

MS/MS of the peptides generated by in-gel digestion was conducted *via* nano-ESI on a Q-TOF2 mass spectrometer (Micromass). The product ions were analyzed with an orthogonal TOF analyzer equipped with a reflector, a micro-channel plate detector and a time-to-digital converter. The data were then processed using a Mass Lynx Windows NT PC system (Micromass). All MS/MS spectra recorded on tryptic peptides derived from spots were searched against protein sequences from NCBInr databases using the MASCOT search program (version 2.1 Matrix Science, Boston, MA, USA). The non-redundant database NCBInr from NCBI is a frequently used protein database for protein identification.

### *In vivo* mouse model and cytometric bead array

C57BL/6 mice were purchased from Orient BIO (Sung-nam, Korea) and maintained in laboratory animal facilities. Six-week-old inbred female mice were used for the experiments. The animal experiments were performed in accordance with the NIH guidelines (USA) for laboratory animal use and care. Inflammation was induced by subcutaneous injection of 100 μg ml^−1^ of LPS (*E. coli* serotype 0111:B4; Sigma). The LPS-stimulated mice were treated with 20 μg, 40 μg or 80 μg of rhTRX by subcutaneous injection.

Using a CBA kit (BD Biosciences, San Diego, CA, USA), the levels of IL-6, IL-10, MCP-1, IFN-γ, TNF-α and IL-12 were quantitatively measured from serum. The results were analyzed with a BD CBA analysis software (BD Biosciences).^32, 33^ Control mice received sterile PBS, and five mice were used for each data point (*n*=5).

### Oil Red O staining

Cryosections on glass slides were fixed with 10% (v/v) formaldehyde in PBS for 1 h at 23 °C, rinsed twice with water and then stained with 0.1% (w/v) Oil Red O in 75% (v/v) isopropanol at 23 °C. After 2 h, the stained cryosections on glass slides were rinsed twice with water to remove unincorporated dye and photographed using a Zeiss AxioVert microscope with phase contrast optics and Hamamatsu digital/video camera.

### Statistical analysis

All data were expressed as means±s.d. Statistical comparisons were made using Student's *t* test or ANOVA coupled with a Fisher's test. A statistically significant difference was defined as *P*<0.05, which is represented by an asterisk in the data presentation.

## Results

### TRX inhibits LPS-stimulated production of ROI and NO

**Figure 1:**
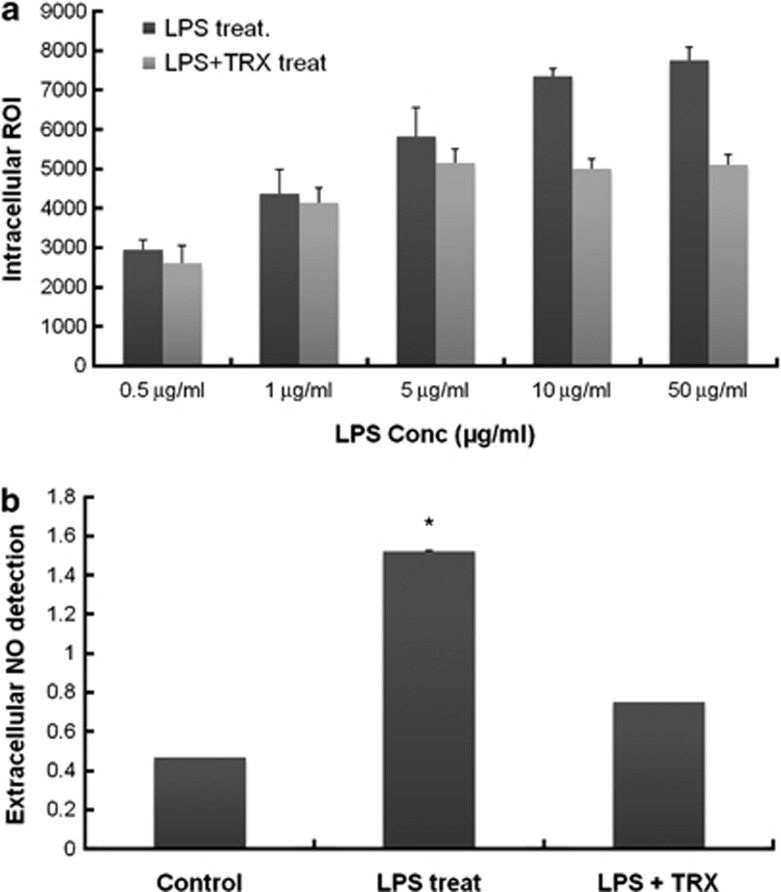
Effect of exogenous rhTRX on production of ROI and NO in LPS-stimulated A375 melanoma cells. (**a**) A375 melanoma cells (3 × 10^5^ per ml) were incubated in medium containing various concentrations of LPS for 4 h and then administered with or without 50 μg ml^−1^ of rhTRX. After 4 h, the cells were collected, washed with PBS and treated with 50 μM of DCFH-DA. The ROI levels were measured using flow cytometry. Data represent the mean±s.d. (**b**) A375 melanoma cells (3 × 10^5^ per ml) were stimulated with 10 μg ml^−1^ of LPS for 4 h and then treated with or without 50 μg ml^−1^ of rhTRX. After 4 h, the nitrite concentration was determined in the supernatant fluid using the Griess assay. The Griess assay data represent the s.e.m. of at least three different experiments (*P⩽0.05 vs controls).

Effects of TRX on oxidative stress and inflammation in skin were investigated by examining the ROI and NO levels in A375 melanoma cells treated with or without rhTRX after exposure to LPS. First, to examine whether rhTRX influences ROI levels, A375 melanoma cells were incubated in medium containing various concentrations of LPS for 4 h followed by incubation with or without 50 μg ml^−1^ of rhTRX. After 4 h, the intracellular ROI level was quantified using a fluorescein derivative DCFH-DA as a redox indicator. A LPS concentration-dependent increase of intracellular ROI was observed in A375 melanoma cells (Figure 1a). In contrast, in cells treated with rhTRX, the increased intracellular ROI level following LPS stimulation decreased, which indicates that exogenous rhTRX treatment diminished the oxidative stress.

LPS also remarkably increased NO production in A375 melanoma cells, which was greatly decreased by rhTRX (Figure 1b). The NO level produced from cells exposed to 10 μg ml^−1^ of LPS for 4 h was 3.25-fold higher than that from control cells. However, treatment with 50 μg ml^−1^ of rhTRX for another 4 h reduced the LPS-stimulated increase in NO by 2.03-fold, which demonstrates the protective effects of rhTRX against inflammation. Taken together, TRX might protect melanocytes from oxidative stress and inflammation-induced damage by attenuating the LPS-stimulated production of ROI and NO.

### TRX attenuates LPS-stimulated changes in protein expression

**Figure 2:**
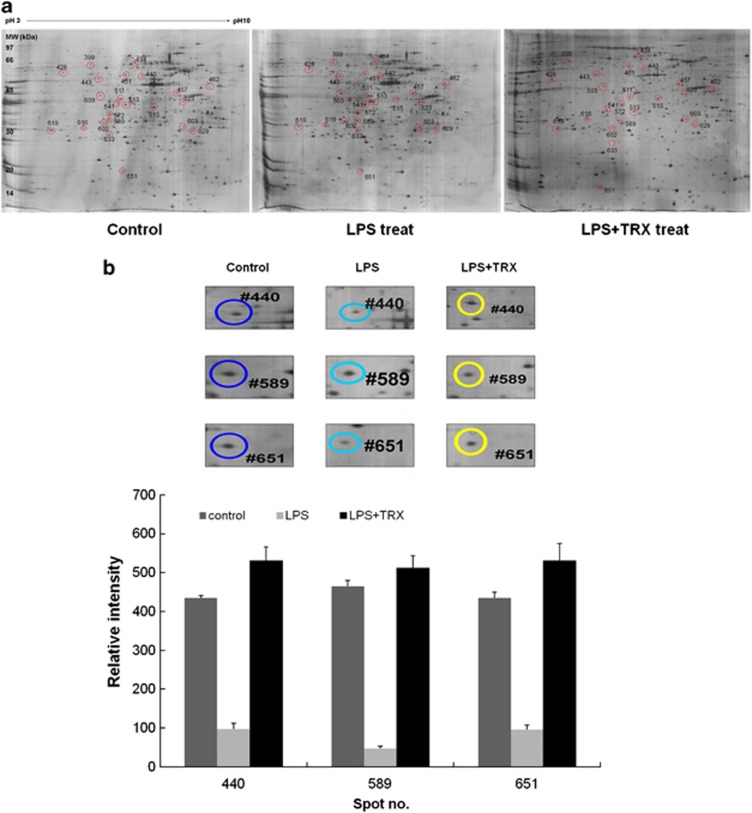
Proteomic analysis of A375 melanoma cells exposed to LPS and then treated with or without rhTRX. A375 melanoma cells were exposed to 10 μg ml^−1^ of LPS for 4 h and subsequently treated with or without 50 μg ml^−1^ of rhTRX for 4 h. Proteins (50 μg) were separated by IEF using 24 cm, pH 3-10 IPG strips and 12.5% homogenous SDS–PAGE. The gels were visualized with silver staining, and their maps were analyzed with an Image Master 2D Elite Software (GE Healthcare, Sweden). (**a**) Representative 2-DE maps. (**b**) Magnified 2-DE maps and relative volume intensity of three differentially expressed spots between A375 melanoma cells treated with or without rhTRX after LPS stimulation. The three proteins were identified as TIP47 (#440), ATP synthase (#589) and thioredoxin (#651) by ESI Q-TOF MS/MS.

As exogenous rhTRX treatment reduced the LPS-stimulated production of ROI and NO (Figure 1), it was highly possible that this treatment affected expression of proteins related to oxidative stress and inflammation. To systematically determine this, we used proteomic techniques. Proteomes in A375 melanoma cells exposed to 10 μg ml^−1^ of LPS for 4 h followed by treatment with or without 50 μg ml^−1^ of rhTRX for 4 h were analyzed using the two-dimensional electrophoresis (2-DE) and compared with in the proteomes of control cells. two-dimensional electrophoresis analysis revealed that exposure of A375 melanoma cells to LPS with or without TRX resulted in marked changes in protein levels compared with control cells (Figure 2a). Among the proteins visualized on the 2-DE gels, 14 spots that were significantly up or downregulated, which showed either 50% downregulation or 100% upregulation compared with those of control cells within the 95% significance level, were selected for identification *via* ESI Q-TOF MS/MS (Tables 1 and 2). Table 1 shows two upregulated and four downregulated proteins in LPS-stimulated A375 melanoma cells, compared with control cells. The upregulated proteins were identified as pyrophosphatase and ubiquitin carboxyl-terminal esterase. The downregulated proteins were cargo selection protein TIP47, prohibitin, ATP synthase and TRX. Table 2 shows five upregulated and three downregulated proteins in A375 melanoma cells stimulated with LPS and then treated with rhTRX, compared with control cells. The differentially expressed proteins included pyrophosphatase, ubiquitin carboxyl-terminal esterase, pyrophosphatase, heterogeneous nuclear ribonucleoprotein and nuclear chloride channel.

Of the proteins that were differentially expressed by LPS stimulation, exogeneous rhTRX significantly reduced the LPS-stimulated changes in the protein expression of cargo selection protein TIP47, ATP synthase and TRX (Figure 2b). Exposure of A375 melanoma cells to LPS resulted in distinctly decreased levels of cargo selection protein TIP47, ATP synthase and TRX, which were completely restored to baseline levels by exogenous rhTRX. On the basis of the previous reports demonstrating that TIP47 and ATP are intimately associated with lipid metabolism,^34, 35, 36, 37, 38, 39^ we concluded that TRX might exert its protective effects on LPS-mediated inflammation at least in part through its ability to modulate a lipid metabolism-dependent pathway by upregulating proteins related to this pathway, such as TIP47 and ATP synthase.

### TRX reduces inflammation-induced lipid accumulation

**Figure 3:**
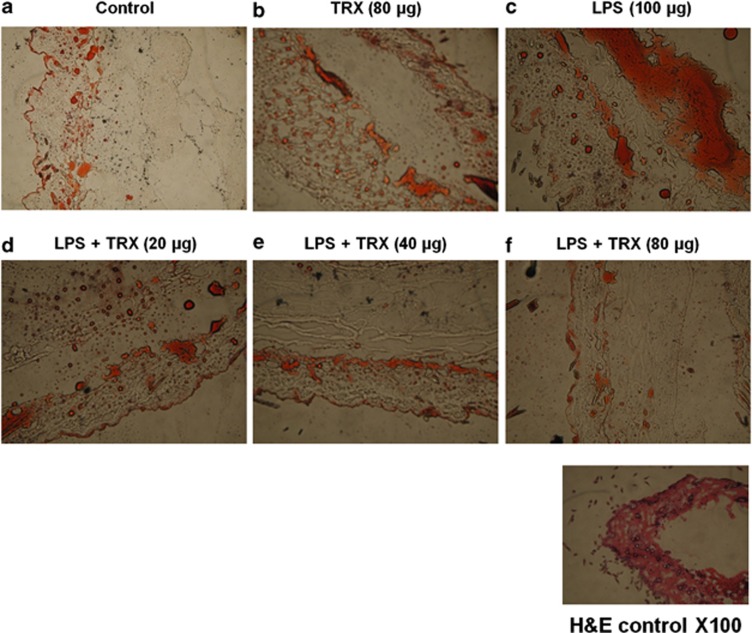
Effect of TRX on inflammation-induced lipid accumulation. C57BL/6 mice were subcutaneously injected with 100 μg of LPS and, after 4 h, with various concentrations of rhTRX. After another 4 h, cryostat sections of the skin were prepared and stained with Oil Red O. The LPS-stimulated mice were treated with 20μg, 40μg, or 80μg of rhTRX by subcutaneous injection (**a**–**f**). Images were acquired at a magnification of × 100.

The Oil Red O stain was used to confirm the results obtained from the 2-DE analysis, suggesting that the protective effects of TRX against inflammation-induced skin cell damage might involve inhibiting alterations in lipid metabolism. A subcutaneous injection of LPS significantly promoted lipid accumulation in the skin of C57BL/6 mice (Figure 3). However, exogenous rhTRX inhibited the effect of LPS in a concentration-dependent manner. As the rhTRX concentration was increased, Oil Red O-stained lipid droplets in the cytoplasm decreased gradually in LPS-injected mice. On the basis of the results shown in Figures 2 and 3, we concluded that TRX protects against inflammation-induced skin cell damage by reducing lipid accumulation.

### TRX decreases inflammatory cytokines

**Figure 4:**
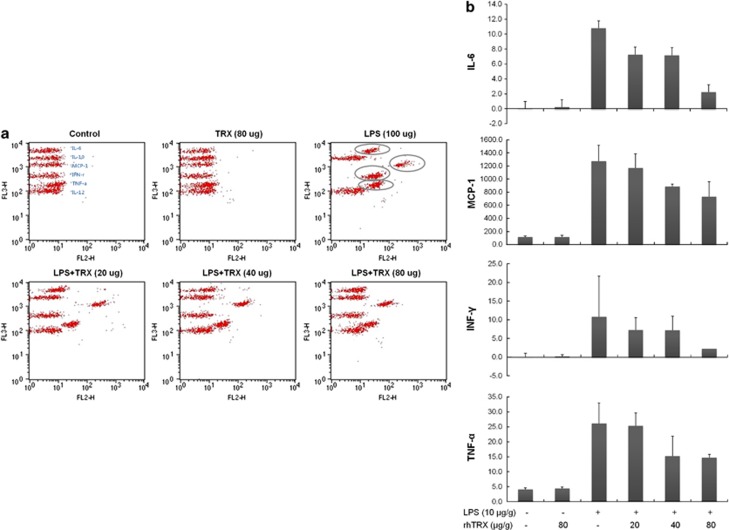
Comparison of inflammatory cytokine levels as determined by CBA (**a**), represented in concentrations of differentially expressed cytokines (**b**). C57BL/6 mice were subcutaneously injected with 10 μg of LPS and, after 4 h, with various concentrations of rhTRX. After another 4 h, the levels of IL-6, IL-10, MCP-1, IFN-γ, TNF-α and IL-12 in serum were quantitatively measured by CBA.

We determined whether skin cell damage after LPS stimulation involves inflammatory cytokines. In these experiments, rhTRX was shown to affect LPS-induced damage. As depicted in the cytometric bead array (CBA) results (Figure 4), inflammatory cytokines IL-6, MCP-1, IFN-γ and TNF-α were significantly increased in LPS-injected mice. However, rhTRX dose dependently reduced the secretion of the inflammatory cytokines stimulated by LPS. Levels of IL-10 and IL-12 were not changed in LPS-injected mice. In addition, mice treated with only rhTRX did not exhibit significant differences in the levels of cytokines examined, which indicates that the inactive network of exogenous rhTRX was rapidly activated in response to bacterial LPS. It should be noted that among the inflammatory cytokines, TNF-α, MCP-1 and IL-6 have also been reported to affect lipid metabolism. Therefore, we concluded that the anti-inflammatory effects of TRX involve decreasing the levels of inflammatory cytokines that alter lipid metabolism.

## Discussion

TRX is an oxidoreductase that contains a dithiol–disulfide active site. Its two cysteines in a CXXC motif contribute to the function of TRX as an antioxidant.^9, 10, 11, 12^ It has been reported that extracellular levels of TRX increase in response to oxidative stress and inflammation,^8, 19^ which indicates the direct involvement of TRX in cellular defense systems. This study also demonstrates the protective effects of TRX against oxidative stress and inflammation by showing that exogenous rhTRX decreased the LPS-stimulated production of ROI and NO in A375 melanoma cells (Figure 1).

After demonstrating that exogenous rhTRX alleviated the inflammation induced by bacterial LPS, it was necessary to identify the proteins associated with this effect, as these proteins could become potential targets for inflammatory studies and drug development. Proteomic analysis was performed to evaluate the protection mechanism of TRX against inflammation-induced skin damage. In these experiments, TRX was found to not only decrease LPS-stimulated production of ROI and NO in A375 melanoma cells but also reduce LPS-induced changes in protein expression (Tables 1 and 2, and Figure 2). In particular, of the differentially expressed proteins by LPS stimulation, exogenous rhTRX significantly attenuated LPS-induced downregulation of cargo selection protein TIP47, ATP synthase and TRX. This suggests a potential link between the ability of TRX to decrease LPS-stimulated production of ROI and NO and its ability to stimulate the expression of three genes encoding cargo selection protein TIP47, ATP synthase and TRX under inflammatory conditions. Inflammation has been recognized as a central component in the pathogenesis of many life-threatening diseases.^40^ One hallmark of inflammation is non-enzymatic oxidation of cellular lipids resulting in the formation of bioactive and toxic products, which modulates inflammation.^40, 41^ An increase in the content of cellular lipids is also known to activate inflammation.^42^ Many studies reported that cellular inflammatory responses rely on diverse factors that determine lipid metabolism. Proteins of the PAT family, including perilipin, adipophilin and TIP47, have been recognized as critical regulators of lipid accumulation.^43^ The PAT proteins are not only associated with lipid droplets but also directly involved in the biogenesis of lipid droplets.^43, 44^ Further, they regulate lipid droplet turnover by modulating lipolysis. Recent studies have shown that downregulation of the PAT proteins results in abnormal lipid droplet metabolism and accumulation, which leads to the development of insulin resistance in obesity.^45^ ATP synthase is also known to be intimately associated with lipid metabolism.^46, 47, 48, 49^ Accordingly, LPS-downregulated expression of TIP47 and ATP synthase demonstrates that LPS induces inflammation by affecting the inflammatory signaling substances such as ROI and NO, as well as by inducing alterations in lipid metabolism. In the same manner, TRX might exert its protective effects on LPS-stimulated cells at least in part through its ability to modulate a lipid metabolism-dependent pathway by upregulating related proteins such as TIP47 and ATP synthase.^35, 36, 37, 38, 39, 50^ The effect of TRX on inflammation-related lipid accumulation in the skin was directly verified by the results of Oil Red O staining, which demonstrated that exogenous rhTRX inhibited lipid accumulation after subcutaneous injection of LPS (Figure 3). These results demonstrate that TIP47 and ATP synthase can be considered early markers of a systemic inflammatory reaction in the pathogenesis of skin diseases and associated metabolic syndromes. As a correlation between inflammation and lipid metabolism was observed, development of treatments that selectively target TIP47 and ATP synthase is likely to be effective to slow the progression of inflammatory skin diseases.

The inflammatory response has been reported to be regulated by a variety of cytokines and lipid mediators. Therefore, the design of new drugs to more selectively and completely inhibit cytokines and lipid mediators has emerged as a rational strategy to improve the efficacy of inflammatory disease treatments. The effects of available therapies on the synthesis of inflammatory cytokines and lipid mediators have also been assessed. As a pleiotropic inflammatory cytokine, TNF-α possesses both growth stimulating and inhibitory properties during inflammation. For instance, TNF-α induces not only neutrophil proliferation but also apoptosis upon binding to the TNF-R55 receptor.^51^ Previous studies have shown that TNF-α is involved in the modulation of many lipid metabolism-related genes.^52^ It has been also shown that the early stage of the metabolic syndrome is marked by a higher level of TNF-α, which is correlated with changes in lipid metabolism and insulin concentrations.^53^ Changes in the level of MCP-1 have been also reported to lead to alterations in lipid metabolism. MCP-1 participates in adipogenesis, and its deficiency is known to prevent high-fat-induced obesity.^54^ IL-6 is also involved in lipid metabolism as well as inflammatory processes.^55^ As depicted in the CBA results (Figure 4), rhTRX dose dependently reduced LPS-stimulated secretion of inflammatory cytokines IL-6, MCP-1, IFN-γ and TNF-α. Considering that IL-6, MCP-1 and TNF-α are potent lipid metabolism regulators in various inflammatory diseases, the results of Figure 4 imply that a correlation among LPS stimulation, lipid accumulation and cytokine secretions exists: LPS exacerbates inflammation at least in part by prompting the secretion of inflammatory cytokines that contribute to disorders of lipid metabolism. These results provide useful information to expand our understanding of how cytokines are regulated in inflammatory responses, which have important implications in the development of effective treatments targeting inflammatory diseases. By contrast, the anti-inflammatory effects of TRX appear to involve decreasing the alterations in lipid metabolism by modulating related cytokines, such as TNF-α, MCP-1, and IL-6. On the basis of the results showing that TRX influences both the expression of TIP47 and ATP synthase and the production of inflammatory cytokines involved in lipid metabolism, further research will be required to support the exciting possibility that a specific correlation exists among TIP47, ATP synthase and those cytokines in the modulation of lipid metabolism during inflammation.

The combined results of this study deepen our understanding of how TRX can protect against inflammatory skin diseases. TRX attenuates inflammation-induced skin cell damage, most likely through decreasing alterations in lipid metabolism by modulating related proteins, such as TIP47 and ATP synthase and cytokines, such as TNF-α, MCP-1 and IL-6. Although further clinical investigations are required to prove the significance and effectiveness of TRX, administration of TRX holds promise as a rational therapeutic strategy to treat inflammation-related disorders of lipid metabolism.

## Figures and Tables

**Table 1:** Identification of the up- and downregulated proteins in LPS-stimulated A375 melanoma cells compared with control A375 melanoma cells using ESI Q-TOF MS/MS

*Spot No.*	*Protein identification*	*Accession No. (gi)*	*MW (Da)*	*pI*	*MOWSE score*^a^	*Queries matched*	*Sequence coverage (%)*	*Up/Down*
541	Pyrophosphatase 1	gi|11056044	33 095	5.54	148	3	11	Up
629	Ubiquitin carboxyl-terminal esterase L3	gi|5174741	26 337	4.84	192	3	22	Up
440	Cargo selection protein	gi|3095186	47 175	5.3	327	7	14	Down
513	Prohibitin	gi|4505773	29 843	5.57	408	7	29	Down
589	ATP synthase, H+ transporting, mitochondrial F0 complex, subunit d isoform a	gi|5453559	18 537	5.21	57	1	10	Down
651	Thioredoxin-like 5	gi|14249348	14 217	5.4	216	4	46	Down

a Score is –10 × log(*P*), where *P* is the absolute probability that the observed match between the experimental data and the database sequence is a random event. The NCBInr database is used through MASCOT searching program (http://www.matrixscience.com/) with ESI-Q-TOF MS/MS data as an input.

**Table 2:** Identification of the up- and down-regulated proteins in LPS-stimulated A375 melanoma cells treated with exogenous rhTRX compared with control A375 melanoma cells using ESI Q-TOF MS/MS

*Spot No.*	*Protein identification*	*Accession no. (gi)*	*MW (Da)*	*pI*	*MOWSE score*^a^	*Queries matched*	*Sequence coverage (%)*	*Up/Down*
381	Zinc-finger protein 259	gi|4508021	51 463	4.66	108	3	9	Up
541	Pyrophosphatase 1	gi|11056044	33 095	5.54	148	3	11	Up
608	Nm23 protein	gi|35068	20 740	7.07	185	5	33	Up
619	Ribosomal protein P2	gi|4506671	11 658	4.42	213	5	69	Up
629	Ubiquitin carboxyl-terminal esterase L3	gi|5174741	26 337	4.84	192	3	22	Up
385	Pyrophosphatase 1	gi|11056044	33 095	5.54	148	3	11	Down
434	Heterogeneous nuclear ribonucleoprotein K transcript variant	gi|59381084	51 312	5.19	183	5	20	Down
511	Nuclear chloride channel	gi|4588526	27 249	5.02	403	7	42	Down

a Score is –10 × log(*P*), where *P* is the absolute probability that the observed match between the experimental data and the database sequence is a random event. The NCBInr database is used through MASCOT searching program (http://www.matrixscience.com/) with ESI-Q-TOF MS/MS data as an input.
